# Potential role of IFN-α in COVID-19 patients and its underlying treatment options

**DOI:** 10.1007/s00253-021-11319-6

**Published:** 2021-05-05

**Authors:** Lei Yang, Jianhui Wang, Pei Hui, Timur O. Yarovinsky, Saiaditya Badeti, Kien Pham, Chen Liu

**Affiliations:** 1 Department of Pathology, Yale School of Medicine, New Haven, CT 06511 USA; 2 Department of Pathology, Rutgers New Jersey Medical School, Newark, NJ 07103 USA

**Keywords:** Coronavirus infection, COVID-19, Interferon-alpha, Treatment, Therapeutic strategies

## Abstract

The coronavirus disease (COVID-19) caused by a severe acute respiratory syndrome coronavirus 2 (SARS-CoV-2) has spread rapidly worldwide. Given that this contagious viral outbreak is still unfolding, it is urgent to understand the pathogenesis of SARS-CoV-2 infection and explore effective treatments to protect patients from developing a severe illness related to COVID-19. Recently, IFN-α has been considered a potential therapeutic strategy to treat COVID-19 disease, mainly because the innate immune system rapidly produces IFN-α as the first line of defense to combat viral infections. However, IFN-α can also play a role in immunoregulatory effects, causing pathogenic damage and uncontrolled inflammatory responses. There are 13 human IFN-α subtypes that bind to the same receptor and induce different interferon-stimulated gene (ISG) expression, regulating various antiviral and immunoregulatory effects. The varying degrees of inflammatory regulations may raise concerns about the possible side effects to enlarge the inflammatory responses, exacerbating the severity of infection. Thus, the analysis of various IFN-α subtype induction during SARS-CoV-2 infection is necessary in exploring the mechanism of COVID-19 pathogenesis. This review summarizes the current understanding of IFN-α in the pathogenesis of respiratory virus diseases and IFN-α based clinical intervention used in SARS-CoV-2 infection and other respiratory virus diseases. Besides, new ideas in selecting suitable IFN-α subtypes or combinations as drug candidates for viral infection treatment will also be discussed.

**Key Points**

*• IFN-α plays an important role in anti-viral and immunoregulatory effects in COVID-19 patients caused by SARS-CoV-2.*

*• The uncontrolled inflammation and disease severity correlated to the diversity of IFN-α subtype induction.*

*• Selecting suitable IFN-α subtypes or combinations as drug candidates will be beneficial for the treatment of patients with COVID-19.*

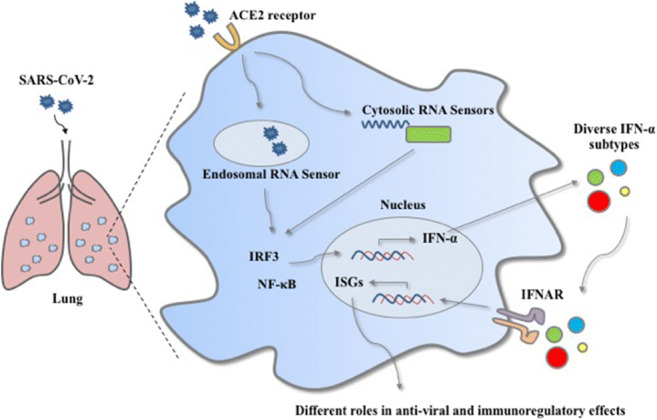

## Introduction

The 2019 coronavirus disease (COVID-19) emergence, caused by severe acute respiratory syndrome coronavirus 2 (SARS-CoV-2), has been deemed a global pandemic since it has swept across 202 countries (Kissler et al. 2020). As of April 1^st^, 2021, SARS-CoV-2 has caused more than 136 million documented infection cases and nearly 3 million deaths worldwide (https://coronavirus.jhu.edu/map.html). COVID-19’s quick emergence and uncontrolled widespread have imposed a continuous threat to humans. Infection with SARS-CoV-2 results in diverse clinical symptoms for COVID-19, ranging from mild to serious respiratory illness. The severe cases were mainly shown as pneumonia and acute respiratory distress syndrome (ARDS), leading to a worse prognosis (Guan et al. 2020). The primary mechanism of ARDS is associated with the uncontrolled systemic inflammatory responses and the release of massive pro-inflammatory cytokines (Conti et al. 2020). This so-called cytokine storm initiates the inflammatory-induced lung injury, causing respiratory failure. Thus, there is an urgent need to better understand the molecular mechanisms of SARS-CoV-2 pathogenesis to explore the potential clinical treatment.

SARS-CoV-2 infection is initiated by the binding of the viral spike proteins to the host cells-expressing angiotensin-converting enzyme 2 (ACE2) receptor (Zhou et al. 2020b), which stimulates the membrane fusion to release the viral RNA. After releasing, the viral genomic RNA or the intermediates during viral replication are recognized by the pattern recognition receptors (PRRs), which will activate downstream signaling pathways to induce type I interferon (IFN-I) production (Li et al. 2020a). The IFN-I molecules then bind to the cell-surface receptor IFN-α and IFN-β receptor (IFNAR), resulting in the transcription of hundreds of interferon-stimulated genes (ISGs) that block the replication and the spread of the virus (Kawai and Akira 2010). Thus, IFN-Is are among the first cytokines produced during viral infection to promote body’s immune responses. In COVID-19 patients, IFN-I signaling is required for ISG induction and the recruitment of pro-inflammatory cells in the lung, indicating its necessary role in host defense against viral infection. Furthermore, several studies have identified that the loss of IFN-I related immunity could cause a severe symptom in the COVID-19 patient (Zhang et al. 2020). This observation indicates that the effective activation of innate immunity, mainly through IFN-I responses and its downstream cascades, is essential to suppress and eliminate viral replication during SARS-CoV-2 infection.

Upon infection, the host mobilizes the innate immune system to rapidly produce IFN-Is, specifically IFN-α and IFN-β, to defend against viral infection. However, several studies demonstrated that the excessive activation of IFN-α signaling pathway might cause the uncontrolled inflammatory responses, which is positively correlated with the disease severity (Channappanavar et al. 2019). Interestingly, all 13 subtypes of human IFN-α bind to the same receptor and induce different downstream ISG expressions (Sutter et al. 2016). The diversity in ISG stimulations may exhibit distinct biological activities in antiviral effects and immune regulatory responses. Accordingly, we may presume that the disease severity of COVID-19 may be related to various IFN-α subtype induction. It has been reported that IFN-α subtypes were differently induced in the high or low pathogenic influenza virus-infected human epithelial cells or mouse, accompanied with the varying degrees of inflammatory responses (Yang et al. 2020). This evidence provides the close connection between the IFN-α subtypes and the uncontrolled inflammatory responses, which may further cause pathological damage to increase disease severity. Additionally, the IFN-α subtype induction analysis paves a way to understand the underlying mechanism of the pathogenesis, and it could further determine specific IFN-α subtype(s) associated with higher antiviral effects and lower immunoregulatory responses. Once identified, the specific IFN-α subtypes, or a combination with other antiviral drugs, could be used as a drug candidate that helps enhance antiviral effects while minimizing immunopathology to the host. This review summarized the current understanding of IFN-α and its mechanism of pathogenesis. New ideas on selecting suitable IFN-α for clinical treatment will also be discussed.

## The origin and transmission of SARS-CoV-2

SARS-CoV-2 was first discovered in the bronchoalveolar lavage fluid (BALF) of three COVID-19 patients from Wuhan Jinyintan Hospital on December 30, 2019 (Zhu et al. 2020). As its rapid transmission, SARS-CoV-2 has swept across 202 countries, shown as a global pandemic (Li et al. 2020c). To date, the person-to-person transmission is the main mechanism which occurs with an infected patient intimately contacting other individuals. The droplets and aerosolized viral particles expelled from the COVID-19-infected individual to nearby individuals through talking, coughing, or sneezing were considered the primary media for the transmission (Ong et al. 2020). Aside from the person-to-person transmission, virus can also be acquired through direct contact with abiotic surfaces (Rothan and Byrareddy 2020), which serve as potential transmission and spread of the SARS-CoV-2 virus. Thus, taking effective transmission control is a strategy for decreasing the infection rate and protecting everyone from an infection.

SARS-CoV-2 is a member of the coronavirus family and has an enveloped positive-stranded RNA genome (Wang et al. 2020c). In the mid-1960s, the coronaviruses (CoVs) were first identified and classified into four distinct subfamilies, α−/β−/γ−/δ-CoV, which were based upon their genotypical and serological characteristics (Guo et al. 2020). Among them, α and β-CoV mainly infect mammals, while γ and δ-CoV mainly infect birds (Guo et al. 2020). Based on the severity of the infection, CoVs can be divided into lower and higher pathogenic subtypes. The lower pathogenic coronaviruses induce a mild infection in the upper and lower respiratory tract such as CoV-229E and CoV-NL63. In comparison, the higher pathogenic coronaviruses infect the lower respiratory tract, causing severe symptoms and even respiratory failures such as the ones found in severe acute respiratory syndrome coronavirus (SARS-CoV) and Middle East respiratory syndrome coronavirus (MERS-CoV) (Channappanavar and Perlman 2017). The current SARS-CoV-2 pandemic is the third coronavirus outbreak occurring in the past 2 decades and shares about 79% and 50% of its genetic sequence with the coronaviruses responsible for the SARS-CoV pandemic in 2002 and MERS-CoV pandemic in 2012 (Wang et al. 2020c; Yin and Wunderink 2018). In the SARS-CoV-2 genetic encoding sequence, there are 27 different proteins, including the spike (S) protein, envelope (E) protein, membrane (M) glycoproteins, and nucleocapsid (N) protein (Wu et al. 2020b). The spike glycoprotein was found to acquire 21 mutations in the binding region, which indicates that this novel coronavirus gradually evolved by adapting to its human hosts through its higher binding power (Forni et al. 2017; Wen et al. 2020). Thus, an urgent need is to better understand this novel SARS-CoV-2 and its host-pathogen biology, which may provide new insights into the effective treatment of patients with COVID-19 and to curtail the current pandemic.

## The entry of SARS-CoV-2

SARS-CoV-2 primarily infects and enters the cells by the viral S protein binding with the host ACE2 receptor (Luan et al. 2020; Wrapp et al. 2020), a membrane-anchored carboxypeptidase highly expressed by airway epithelial and type I and II alveolar epithelial cells (Kim et al. 2020; Zhou et al. 2020a). This receptor is also highly expressed on the cell surface of many tissues and organs such as the heart, kidney, intestine, and blood vessels (Zhou et al. 2020a), which may explain why some COVID-19 patients (46%) experience renal, gastrointestinal, and cardiovascular problems (Gu et al. 2020). Interestingly, ACE2 mRNA level also appear to be regulated by multiple factors, including age and tobacco smoke (Busnadiego et al. 2020). Since ACE2 is the receptor of the S protein which mediates viral invasion, the soluble form of ACE2 might be considered a potential therapeutic strategy to prevent the SARS-CoV2 infection (Batlle et al. 2020; Sun et al. 2020). Some studies have focused on the monkey kidney’s cell line Vero-E6 which have shown that soluble ACE2 could block SARS-CoV replication by binding to its viral particles and preventing them from entering into the cells (Li et al. 2003). Upon the S protein’s receptor recognition, the viral entry into the target cells also requires S protein priming by cellular proteases, the human transmembrane protease serine 2 (TMPRSS2). It can cleave at the S1/S2 boundary, and then the S2 subunit allows for the fusion of viral and cellular membranes to enter into the cells (Hoffmann et al. 2020; Xia et al. 2020; Yang and Shen 2020). After SARS-CoV-2 enters the cells, it subsequently releases its viral RNA into the cytoplasm and translates by its host ribosomes to produce viral replicative enzymes which generate new RNA genomes. Finally, the viral RNAs direct these necessary components to synthesize and assemble new viral particles (Astuti and Ysrafil 2020).

In addition to recognizing and binding the ACE2 receptor of SARS-CoV-2, several studies have found a novel invasion route through CD147 and S protein, by using co-immunoprecipitation and ELISA methods to prove the binding of CD147 and S protein (Wang et al. 2020d). In the same study, researchers have also shown that an anti-CD147 humanized antibody could significantly inhibit the virus from invading the host cells (Wang et al. 2020d), which indicates anti-CD147’s effective blocking and its ability to reduce SARS-CoV-2 viral infection. Thus, the novel route of CD147-S protein could provide a critical target for developing specific antiviral drugs.

## The pathogenesis of SARS-CoV-2

When an individual contracts the SARS-CoV-2 virus, the patient may experience a wide range of symptoms such as fever (88.7%), cough (67.8%), fatigue (38.1%) ranging from mild to moderate, and severe pneumonia (15%) in several cases (Guan et al. 2020; Wang et al. 2020b). In general, elderly individuals and those who suffer from underlying conditions are susceptible to infection and ultimately experience serious outcomes (Guo et al. 2020). The median age of COVID-19 patients is 47 years old which ranges from 35 to 58 years old (Guan et al. 2020). Characteristically, severe pneumonia is often associated with rapid virus replication, massive inflammatory cell infiltration, and elevated pro-inflammatory cytokine/chemokine responses. These symptoms may cause acute lung injury (ALI) and even a worse acute respiratory distress syndrome (ARDS). Currently, ARDS is considered the leading cause of death in patients who suffer from COVID-19 (Chen et al. 2020; Huang et al. 2020).

For patients with severe COVID-19 disease, the primary concern is their uncontrolled systemic inflammatory responses caused by a massive production of pro-inflammatory cytokines, including IL-6, IP-10, MCP-1, MIP-1α, and TNF-α, with diverse pro-inflammatory roles (Conti et al. 2020; Huang et al. 2020). The excessive and unbalanced pro-inflammation cytokine production causes a “cytokine storm syndrome” which may contribute to a significant organ damage and pathological injuries (Ruan et al. 2020; Tian et al. 2020; Wu et al. 2020a; Xu et al. 2020). The lung injury of a SARS-CoV-2 patient demonstrates bilateral diffuse alveolar damage and the presence of interstitial mononuclear inflammatory infiltration. Such alveolar damage and infiltration are composed of lymphocytes and multinucleated syncytial cells in intra-alveolar spaces, consistent with viral cytopathic-like changes. Such lung injuries may be asymmetric: one side of the lung may show evidence of the desquamation of pneumocytes and hyaline membrane formation, while the other side may display pulmonary edema with a hyaline membrane formation indicating the development of ARDS (Xu et al. 2020). Such pulmonary injuries have also been seen in patients with SARS-CoV and MERS-CoV infection (Ding et al. 2003; Ng et al. 2016). Taken together, the cytokine storm is a common feature in severe COVID-19 infection and responsible for the initiation of inflammatory-induced lung injury, leading to ARDS and respiratory failure. Accordingly, anti-cytokine therapy such as IL-6, TNF-α, and IL-1 antagonists has been suggested as therapeutic approach in the alleviation of hyper-inflammatory response in patients with SARS-CoV-2 infection (Roshanravan et al. 2020).

## Innate immune responses to SARS-CoV-2 infection

The S proteins on SARS-CoV-2’s surface initiate the cellular infection by binding to the host cells via the ACE2 receptor, which in turn stimulates membrane fusion and releases viral RNA. After this release, the viral genomic RNA or dsRNA intermediates are detected by PRRs as pathogen-associated molecular patterns (PAMPs), mainly RIG-I-like receptors (RLRs) and Toll-like receptors (TLRs) (Alexopoulou et al. 2001; Li et al. 2020a; Wu and Chen 2013), which mediate the related downstream signaling pathways to control and eliminate the virus infection.

## RLRs-dependent antiviral signaling pathway

RLRs, including the H family members RIG-I (DDX58), MDA5 (IFIH), and LGP2 and RIG-I and MDA5, are mainly responsible for viral recognition in the cytoplasm (Wu et al. 2013; Yoo et al. 2014). RIG-I directly recognizes and binds to viral 5′-PPP RNA and short dsRNA, while MDA5 senses long dsRNA (Kowalinski et al. 2011; Nikonov et al. 2013). After the recognition, RIG-I and MDA5 converge on the mitochondrial adaptor protein, including mitochondrial antiviral signaling protein (MAVS), interferon-B promoter stimulator 1 (IPS-1), or virus-induced signaling adaptor (VISA), which mediate the signaling cascades (Lee et al. 2018; Tao et al. 2019; Xu et al. 2018). The adaptor proteins trigger TBK1/IKKE and IKKα/IKKβ through TRAFs to mediate the activation of transcription factors (Clément et al. 2008), such as interferon regulatory factor 3 (IRF3) and interferon regulatory factor 7 (IRF7). Upon phosphorylation and activation of IRF3 and IRF7, they are translocated to the nucleus and stimulate IFN-I expression, particularly IFN-α and IFN-β, which initiates the subsequent downstream signaling pathways through the autocrine and paracrine to induce the expression of ISGs and exerts antiviral and immunoregulatory effects.

## TLRs-dependent antiviral signaling pathway

TLRs are essential PRRs for recognizing viral components or replicating intermediates during an infection. TLR3, TLR7, TLR8, and TLR9 sense viral nucleic acid in the endosome, while TLR2 and TLR4 recognize viral proteins on the cell surface (Arpaia and Barton 2011; Wu et al. 2013). After the recognition, the activated TLRs combine with the adaptor molecule MyD88 and TRIF leading to the activation of the downstream signaling cascade such as IRF3, IRF7, and NK-kB, which are accompanied by their nuclear translocation. This subsequently leads to a further induced expression of IFN-Is and a series of pro-inflammatory cytokines (Arpaia and Barton 2011; Kawai and Akira 2010; Kostoula et al. 2019; Spiegel et al. 2005). It has been shown that COVID-19 patients have an enhanced activity of multiple IRFs which may aid in the occurrence of an IFN-I related immune response for controlling and eliminating the viral infection (Liao et al. 2020).

## The role of IFN-I related signaling pathway in SARS-CoV-2 infection

**Fig. 1:**
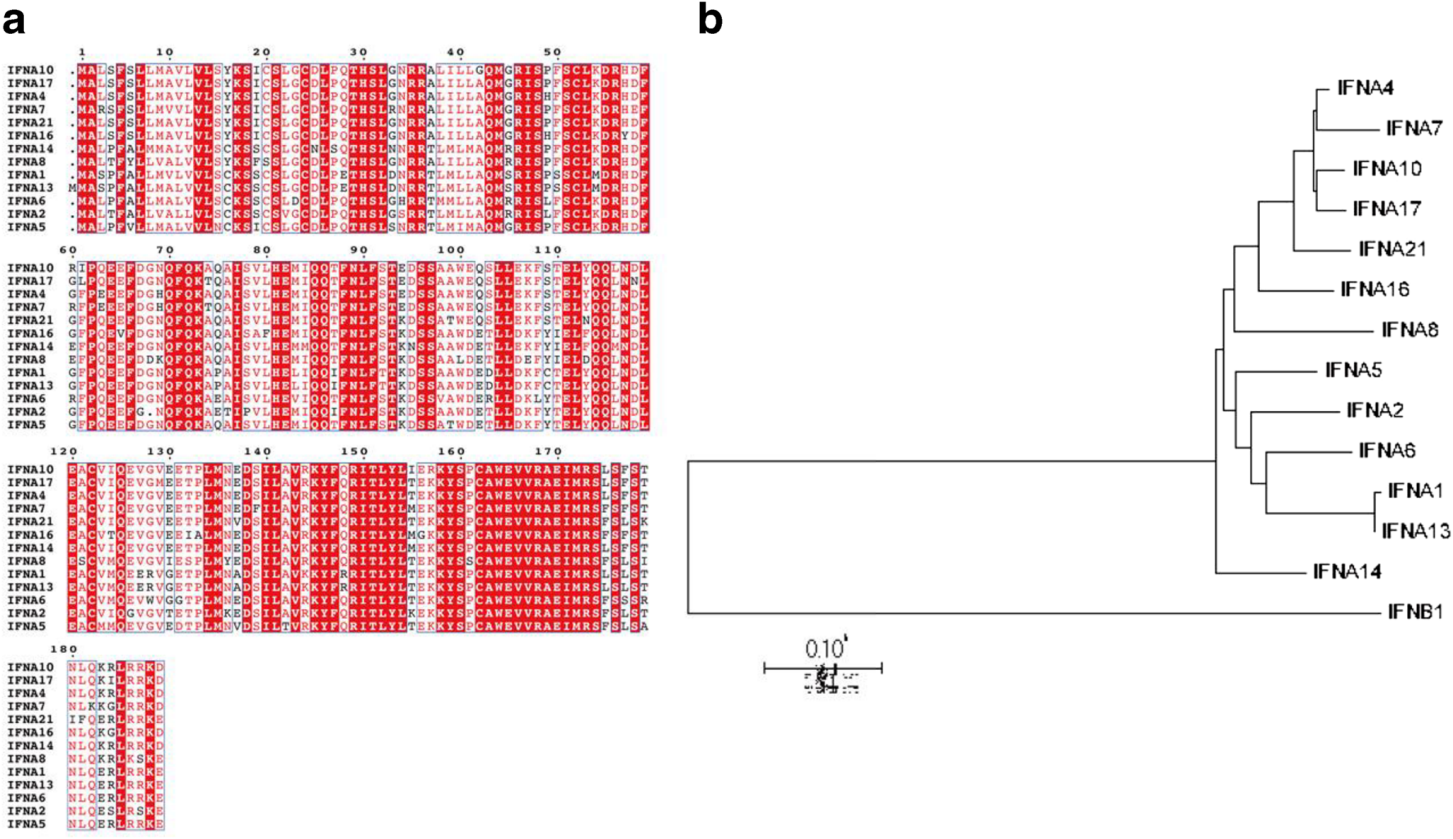
Analysis of various human IFN-α subtypes. **a** The comparison of amino acid sequence. **b** Phylogenetic analysis

When SARS-CoV-2 invades the host, PRRs initially recognize the viral nucleic acid and thus induce a series of signaling cascades to promote the synthesis of IFN-Is, which plays a significant role in antiviral defense for limiting the spread of the virus (Li et al. 2020a). IFNs are critical effectors associated with the activation of both innate and adaptive immune responses against viruses and other microbial infections (Wang and Fish 2019). IFNs are classified as type I (-α, -β, -δ, -ε, -ζ, -κ, -τ, and -ω), II (-γ), and III IFNs (-λ1, -λ2, -λ3), based on the different binding receptors (Hoffmann et al. 2015). Among them, IFN-Is are highlighted for their effectiveness to limit the viral spread (Isaacs and Lindenmann 1957) and involvement in mediating immunoregulatory effects. Noticeably, IFN-α exists with 13 human subtypes sharing similar sequences and structures (Hoffmann et al. 2015; Schreiber and Piehler 2015), and they have a strong correlation with each other (Fig. 1). Additionally, all IFN-α subtypes bind to the same receptor complex IFN-α and IFN-β receptor (IFNAR) which induces different downstream ISG expressions (Sutter et al. 2016). This differential induction may likely be due to the nature of its structural properties, e.g., individual amino acids at specific positions, or the difference in their binding affinity to both receptor subunits of IFNAR (Lavoie et al. 2011), which allows them to play different roles in viral infection.

During SARS-CoV-2 infection, the host mobilizes the innate immune system to rapidly produce IFN-Is as the first defense mechanism for exerting antiviral effects to protect itself from a viral infection (Cao et al. 2019). The produced IFN-Is via the common IFNAR on epithelial and immune cells initiate the Janus kinase signal transducer and activator of the transcription (JAK-STAT) signaling pathway. Following this receptor activation, the transcription factor STAT1 and STAT2 are phosphorylated by Janus protein tyrosine kinases JAK1 and TYK2. The phosphorylated STAT1 and STAT2 are associated with IRF9 to form a complex, IFN-stimulated gene factor 3 (ISGF3). ISGF3 subsequently enters the nucleus to stimulate the transcription of numerous ISGs by binding to the IFN-stimulated response element (ISRE) sequences located in the promotors of target genes (Fish and Platanias 2014; Schneider et al. 2014). The broad range and diverse ISG production could induce an antiviral state in the host to defend itself against viral infection. According to a heat map analysis, several ISGs are upregulated during a SARS-CoV-2 infection, such as IRF7, STAT2, and MX2 (Benjamin et al. 2020), which may help suppress and eliminate viral replication during SARS-CoV-2 infection.

Meanwhile, several studies demonstrated that individuals with insufficient IFN-I production in their immunity are more vulnerable to SARS-CoV-2 infection, indicating the importance of effective IFN-I related antiviral responses during infection. Using gene analysis, the author observed the patients with severe COVID-19 have mutations in genes of TLR3 and IRF7, involved in the regulation of IFN-Is (Zhang et al. 2020). This observation suggests the impaired IFN-I signaling pathways that are correlated with the severity of infection. In addition, a loss-of-function mutation in the TLR7 gene, related to PRR in IFN-I pathways, was also found in four young men with severe COVID-19 illness (Van Der Made et al. 2020), associated with a persistent viral load. Furthermore, high titers of neutralizing autoantibodies against IFN-α2 were detected in about 10% of patients with severe COVID-19 pneumonia, when compared to the asymptomatic or mild phenotype individuals (Zhang et al. 2020). Collectively, these studies suggest that the effective innate immune responses, mainly IFN-I responses and their downstream cascades, are essential for antiviral defense against viral infection.

## IFN-α and COVID-19

IFN-Is have essential roles in protecting the lung from SARS-CoV-2 spreading. In contrast, they have also been critical factors in initiating lung inflammatory responses by inducing the recruitment and activation of immune cells and the secretion of inflammatory cytokines and chemokines. Examples of such immune regulations are chemokine inductions of CCL2 and CXCL10, which play important roles in recruiting monocytes/macrophages, T cells, NK cells, and neutrophils and therefore directly initiate inflammation in the lung (Goritzka et al. 2014). Accordingly, if excessively activated, IFN-I signaling pathways could result in uncontrolled inflammatory responses that exacerbate lung tissue damage, considering as one of the major complications in COVID-19 severity. It was demonstrated that, in the BALF of COVID-19 patients, the increased expression of IFN-α and IFN-β correlates with the morbidity (Broggi et al. 2020), suggesting the potential pathogenesis of IFN-Is during SARS-CoV-2 infection. The same observation was also found in the blood of the severe and mild COVID-19 patients (Lee et al. 2020). Therefore, IFN-I related responses likely play a pivotal role in exacerbating inflammation in severe COVID-19 patients.

Apart from the uncontrolled inflammatory responses, another possible pathogenesis role of IFN-α is the induction of ACE2 in primary human upper airway basal cells (Ziegler et al. 2020). ACE2, the receptor for SARS-CoV-2, was shown to be stimulated by IFN-α. By adopting this potential strategy, SARS-CoV-2 might exploit the ACE2-mediated tissue-protective response to provide other cellular targets for an entry to increase viral infectivity. Arising concerns is about the possibility of ACE2-inducing side effects due to high IFN-α expression in the patients with COVID-19. Therefore, effective therapeutics to control the appropriate level of the IFN-α pathway, not too much and not too less, may reduce the severity of infection while preventing unexpected side effects that may arise with the IFN-α treatment.

There are 13 IFN-α subtypes in human with similar sequences and structures. All IFN-α subtypes bind to the same receptors that induce the production of different ISGs, resulting in diverse biological activities of antiviral effects and immune regulations. The differences could make various IFN-α subtypes induce different intensities of inflammatory cytokines, such as CXCL10. High CXCL10 production could cause excessive recruitment of neutrophils to infectious sites, leading to uncontrolled inflammatory responses. Thus, the analysis of diverse IFN-α subtype induction might be a way to explain the difference in inflammation level. By analyzing this aspect, we might uncover if an induction of the specific IFN-α subtype could be used as an indicator to diagnose the severity of patients with COVID-19. Indeed, the correlation between various IFN-α subtypes and viral pathogenesis was demonstrated in influenza virus-infected human respiratory epithelial cells. Following the analysis of IFN-α subtype cDNA library, IFN-α1, IFN-α6, IFN-α14, and IFN-α16 were found to be more dominantly induced in higher pathogenic influenza A virus (IAV)-infected human respiratory epithelial cells, whereas IFN-α5, IFN-α8, and IFN-α21 were mainly expressed in lower pathogenic influenza B virus (IBV) infection cells (Yang et al. 2020). This observation suggests that IFN-α1, IFN-α6, IFN-α14, and IFN-α16 may have a connection with the higher inflammatory responses, causing more severe clinical symptoms, indicating the close association of diverse IFN-α subtype distribution and the infection severity. Compared to the influenza virus, it is worth to consider the connection in patients with COVID-19. Addressing this question may provide a better understanding of diverse IFN-α subtype induction during SARS-CoV-2 infection to explore underlying mechanism of pathogenesis and a possibility of whether the specific IFN-α subtype induction could be used as an indicator to reflect the infection severity. Also, genetic variants of SARS-CoV-2 have been identified from the original strain in different countries of the world, associated with mild or severe outcome (Voss et al. 2020). Accordingly, diverse IFN-α subtype induction in different SARS-CoV-2 variant infection should also be considered a way to reveal the underlying mechanism of disease severity. Up to date, there is minimal evidence for the relationship between immunological features of IFN-α subtypes and COVID-19 severity, which need to be further explored in the future.

## The role of IFN-α in the treatment of SARS-CoV-2 infection

In contact with a pathogen’s infection, the host immediately mobilizes the innate immune system to rapidly produce IFN-α to defend itself against viral infection as the first line of defense. By inducing antiviral responses, IFN-α alone or in combination with other drugs should be considered the best drug candidates for the treatment of SARS-CoV-2 infection. Clinically, IFN-α, combined with lopinavir/ritonavir (LPV/r), has already been tested in patients with COVID-19. Compared to the patients administered with oral LPV/r, the patients receiving a subcutaneous injection of IFN-α2b combined with LPV/r have a shorter length of hospitalization with an associated enhancement of viral clearance (Wang et al. 2020a). Additionally, administration of IFN-α2b with or without arbidol significantly reduced the duration of detectable virus in the upper respiratory tract and the level of inflammatory cytokine IL-6 in the blood (Zhou et al. 2020b). Clinical studies in Cuba also confirmed the therapeutic effectiveness of IFN-α2b against COVID-19 (Pereda et al. 2020). In the same token, patients who suffer from severe MERS-CoV infection treated with a combination of IFN-α2a and ribavirin experienced a significantly improved survival rate of 14 days (Saad et al. 2015). These findings indicate that IFN-I treatment should be considered an effective therapy in COVID-19 patients.

Although IFN-α could boost broad-spectrum antiviral responses, conversely, it can assert immunoregulatory effects, which may cause pathogenic effects in some cases. Any imbalance implement of IFN-Is would impact the clinical outcome of COVID-19 and other co-existing diseases. As previously reported, MERS-CoV patients receiving IFN-α2a treatment failed to improve their survival rate (Shalhoub et al. 2015). The same outcome was also observed with MERS-CoV-infected patients who received IFN-α2b treatment (Al-Tawfiq et al. 2014). The administration of IFN-I within 1 day after MERS-CoV infection protected mice from lethal infection, whereas the delayed IFN-I treatment failed to effectively inhibit virus replication, accompanied by an enhanced pro-inflammatory cytokine expression and an increased infiltration of neutrophils in the lungs, resulting in fatal pneumonia (Channappanavar et al. 2019). These findings may suggest that the IFN-I administration aggravated the severity of the infection rather than exerting antiviral effects, and the other factors, e.g., administration time and dose, should be included for consideration in any treatment options. Thus, it is imperative to evaluate the beneficial-versus-detrimental effects of IFN-I therapy for COVID-19 patients with divergent disease severity in clinic application. Similar results were also observed in influenza virus-infected mice that the treatment of IFN-α could cause immunopathology, leading to the increased morbidity of the mice (Davidson et al. 2014). Also, recent studies indicate that SARS-CoV-2-infected mice had IFN-Is that failed to control SARS-CoV2 replication but drive pathologic responses (Benjamin et al. 2020). Besides the antiviral effects, the pleiotropic cytokine IFN-α also plays a vital role in immunoregulatory effects, which may cause damage and increase the severity of the infection. Altogether, it is critical to balance antiviral and immunoregulatory effects when applying IFN-α therapy to treat COVID-19.

## Treatment of COVID-19 with suitable IFN-α subtypes

Many potential SARS-CoV-2 treatment approaches have been evaluated in several ongoing studies, such as the inhibition of SARS-CoV-2 fusion/entry, the disruption of replication, the suppression of an excessive inflammatory response, as well as convalescent plasma treatment and vaccines (Li et al. 2020b). Remdesivir (RDV), the RNA-dependent polymerase (RdRp) activity inhibitor nucleotide analog, interferes with the synthesis of new viral RNA by chain termination. It was the first approved medicine against COVID-19 in the European Union and then in the USA, India, and Japan for emergency use to treat COVID-19 patients with severe symptom (Kmietowicz 2020). Dexamethasone, a corticosteroid, has also been found to improve survival in hospitalized COVID-19 patients who require supplemental oxygen (Group 2020). In terms of SARS-CoV-2 monoclonal antibody therapy, a combination of bamlanivimab and etesevimab was also used as the options for the mild to moderate COVID-19 patients. The primary function of bamlanivimab and etesevimab is to specifically bind to different but overlapping sites on the spike protein of SARS-CoV-2, which could block the viral attachment and entry. Although many potential methods are implemented in clinical treatment, we still need to understand the underlying mechanism and explore novel therapeutic interventions for coronavirus infection.

To date, the speedy replicating rate of the virus and the heightened virus-associated inflammatory responses are the most critical contributors to the development of severe pneumonia in patients with SARS-CoV-2 infection (Qin et al. 2020). There is no doubt that the antiviral agents with lower immunoregulatory effects are necessary to treat the COVID-19 disease (Conti et al. 2020; Wang et al. 2020e). When binding to the same receptor IFNAR, diverse IFN-α subtypes stimulate different downstream ISG expression, allowing them to play multiple roles in regulating antiviral and immunoregulatory effects. Therefore, selecting appropriate IFN-α subtypes with higher antiviral effects and lower immunoregulatory functions will benefit the clinical treatment for SARS-CoV-2 infection. Regarding the various IFN-α subtypes, many studies have investigated the altering degrees of antiviral potency and immunoregulatory effects in response to a viral infection (Thomas et al. 2011). Compared to IFN-α2, IFN-α5, and IFN-α6, the treatment of IFN-α1, IFN-α4, IFN-α9, and IFN-α11 in the Friend retrovirus (FV)-infected mice could significantly reduce the viral load (Gerlach et al. 2009; Kathrin et al. 2012). In addition, immunization with IFN-α5 and IFN-α6 subtypes could effectively reduce the viral load in the lungs of BALB/c mice that are challenged with H1N1 IAV, while the vaccine containing IFN-α1 was less effective (James et al. 2007). These findings suggest that diverse IFN-α subtypes show various antiviral effects to different viral infections. Accordingly, it is worth to compare the antiviral effects of all IFN-α subtypes on SARS-CoV-2’s response to better understand the correlation between various IFN-α subtypes and antiviral effects. This evidence will provide an insight for selecting the best drug candidate for effective treatment of COVID-19.

Besides the antiviral effects, the lower immunoregulatory responses induced by different IFN-α subtypes will also be an indicator for selection. Since IFN-α could persuade CXCL10 to recruit neutrophils to the infectious site and control its viral dissemination (Ichikawa et al. 2013; Zhu et al. 2018), the infiltrated neutrophils, if excessively, may cause pathological damage to the respiratory epithelium, which would be detrimental to the host. Thus, CXCL10 induction could be used by various IFN-α subtypes as an indicator to distinguish the magnitude of immunoregulatory effects. Indeed, the relationship between different IFN-α subtypes and CXCL10 production has been previously investigated in human dendritic cells, in which IFN-α2 and IFN-α21 could stimulate the expression of CXCL10, whereas IFN-α1 has no impact (James et al. 2007). These findings suggest that diverse IFN-α subtypes may exhibit different immunoregulatory effects, such as CXCL10 inducibility. The varying degrees of inflammatory regulations induced by different IFN-α subtypes may raise a concern about the possible side effects to enlarge the inflammatory responses, exacerbating the severity of infection. Therefore, the specific IFN-α subtypes, with higher antiviral effects and lower immunoregulatory effects, will be selecting as potential drug candidates for COVID-19 patients.

Another relevant factor that needs to be mentioned is the induction of ACE2, which was recognized as an ISG. It may raise a controversial question for the suitability of IFN-I treatment in patients with COVID-19 because of the possibility that IFN-stimulated induction of ACE2 might increase the entry of SARS-CoV-2. Although interferon could enhance ACE2 expression and surface level, several studies demonstrated that the antiviral action of IFN-Is against SARS-CoV-2 counterbalances any proviral effects of ACE2 induction and subsequently restrict the virus spread (Busnadiego et al. 2020). Thus, ACE2 induction is not the determent factor to be considered. Meanwhile, we could also compare and analyze the diverse IFN-α subtypes induced variability in ACE2 expression levels, and this comparison would provide evidence for us to choose the specific IFN-α subtype to control the infection rate.

Although IFN-α2a and IFN-α2b were respectively approved for the treatment of HBV and HCV infections, they may not be suitable for treating SARS-CoV-2 infection (Li and Clercq 2020; Paul et al. 2015). The principle of selecting practical IFN-α subtypes for the treatment of SARS-CoV-2 is the balance of the antiviral effects and immunoregulatory functions. Through this criterion, we could choose the specific IFN-α subtypes with higher antiviral activity and lower immunoregulation to explore as the novel therapeutic interventions for COVID-19 treatment. Alternatively, a combination of missing “in-action” IFN-α subtypes with a potential increase in antiviral efficiency could be a worth-considering approach to maximize the antiviral effect of IFN-α treatment for COVID-19 patients. Except for the diverse IFN-α subtypes, another type I IFN member, IFN-β, also shows promising results in clinical trials, which may benefit a potentially combined treatment option. A randomized and double-blind phase 2 pilot trial conducted in the UK evaluated the inhaled interferon beta-1a in patients with COVID-19. Compared to the placebo group, the patients receiving nebulized IFN-β-1a had decreased the odds of developing severe disease and shown significant clinical improvement (Monk et al. 2021), suggesting that IFN-β-1a therapy may accelerate the recovery process of COVID-19 patients. Besides its apparent clinical effects, we should carefully consider the inflammatory responses caused by IFN-β in critically ill COVID-19 patients. Furthermore, it is worth to evaluate the role of IFN-β-1a on inflammatory responses and virologic effects and consequently to assess the potential risks and underlying application as a combined treatment with several specific IFN-α subtypes.
